# Laboratory evaluation of stable isotope labeling of *Culicoides* (Diptera: Ceratopogonidae) for adult dispersal studies

**DOI:** 10.1186/s13071-019-3671-9

**Published:** 2019-08-22

**Authors:** Emily G. McDermott, Bradley A. Mullens, Christie E. Mayo, E. Brendan Roark, Christopher R. Maupin, Alec C. Gerry, Gabriel L. Hamer

**Affiliations:** 1 Present Address: Entomology Branch, Walter Reed Army Institute of Research, Silver Spring, MD 20910 USA; 2 Department of Entomology, University of California-Riverside, Riverside, CA 92521 USA; 3 Department of Microbiology, Immunology and Pathology, Colorado State University, Fort Collins, CO 80523 USA; 4 Stable Isotope Geosciences Facility, Department of Geography, Texas A&M University, College Station, TX 77843 USA; 5 Department of Entomology, Texas A&M University, College Station, TX 77843 USA

**Keywords:** *Culicoides sonorensis*, Stable isotopes, Dispersal, Mark-recapture, Bluetongue virus, Epizootic hemorrhagic disease virus

## Abstract

**Background:**

Stable isotope labeling is a promising method for use in insect mark-capture and dispersal studies. *Culicoides* biting midges, which transmit several important animal pathogens, including bluetongue virus (BTV) and epizootic hemorrhagic disease virus (EHDV), are small flies that develop in various semi-aquatic habitats. Previous *Culicoides* dispersal studies have suffered from the limitations of other labeling techniques, and an inability to definitively connect collected adult midges to specific immature development sites.

**Results:**

Adult *C. sonorensis* were successfully labeled with ^13^C and ^15^N stable isotopes as larvae developing in a semi-aquatic mud substrate in the laboratory. High and low-dose isotope treatments for both elements significantly enriched midges above the background isotope levels of unenriched controls. Enrichment had no effect on *C. sonorensis* survival, though a slight (~ 5 day) delay in emergence was observed, and there was no significant effect of pool size on ^13^C or ^15^N enrichment levels.

**Conclusions:**

Stable isotope labeling is life-long, and does not interfere with natural insect behaviors. Stable isotope enrichment using ^13^C or ^15^N shows promise for *Culicoides* dispersal studies in the field. This method can be used to identify adult dispersal from larval source habitat where a midge developed. It may be possible to detect a single enriched midge in a pool of unenriched individuals, though further testing is needed to confirm the sensitivity of this method.

## Background

Knowledge of *Culicoides* dispersal is critical to understand the transmission of pathogens like bluetongue virus (BTV) and epizootic hemorrhagic disease virus (EHDV) between farms. Most studies that have attempted to model *Culicoides* long-distance movement have relied on air current data, with the assumption that infected vectors move across significant distances by wind [1–4]. *Culicoides* biting midges are small (1.0–2.5 mm in length) [5], and are not thought to be strong fliers, though in some instances midges have been recovered several kilometers from a known release point, regardless of wind direction [6, 7]. In instances of long-distance migration, it becomes difficult, if not impossible to link adults in host-seeking areas to larval habitat of origin, unless a suitable marking technique is used [2, 8, 9].

In traditional mark-recapture studies, insects (usually adults) are labeled in a way such that their initial location is known, and can later be identified in collections from other areas to determine a linear estimate of movement from the initial area. Several methods have been used to label *Culicoides* for such studies in both the field and laboratory, including radioactive isotopes [10], fluorescent dusts [7, 11], ingestion of dyes [12], ingestion of rubidium from a marked vertebrate host [13], and immunomarking [14]. These methods require the collection of large numbers of insects initially, because the rate of recapture is often extremely low. For example, Kluiters et al. recovered only 0.02% of the over 61,000 *Culicoides* that were originally labeled in that study [7], although Brenner et al. recovered 14% of labeled females using CO_2_-baited traps in a host-poor desert environment [6]. Typical recapture rates are more often 1–5% [11, 15]. Most *Culicoides* spp. cannot be reared in the laboratory for release, and capturing and labeling the number of insects needed to achieve acceptable recapture rates from the field is unfeasible for most marking technologies. Additionally, the marking technique itself has the potential to impact survival and/or behavior (e.g. fluorescent dust) [16]. The ideal labeling method for these studies should require minimal labor, mark insects without interfering with their natural behaviors, not affect insect survival, be cost effective, and be lifelong [17].

Stable isotopes are naturally occurring, non-radioactive forms of elements in the environment. Previous studies have demonstrated that enriching aquatic habitats with stable isotopes resulted in emerged insects with isotope levels above natural background levels, indicating that stable isotope labeling can be a means of marking insects for dispersal studies [18–20]. Marking the immature environment, rather than collected adults provides uniquely valuable information on adult dispersal from a known development location, resulting in a more precise and accurate estimate of natural movement. Previous work showed that *Culex* mosquitoes (Diptera: Culicidae) could be successfully labeled as larvae in both the laboratory and field using ^15^N-labeled potassium nitrate (KNO_3_) and ^13^C-labeled glucose added to the development water, and that enrichment was detectable in emerged adults up to at least 55 days post-emergence [21]. While mosquitoes have completely aquatic development, larval development in the important *Culicoides* vector species is mainly semi-aquatic or terrestrial [22], and the ability to enrich these habitats using stable isotopes is unknown. *Culicoides sonorensis* Wirth & Jones is the primary North American BTV vector, and lays its eggs on mud at the shallow edges of organically enriched aquatic habitats (e.g. dairy wastewater ponds) [23]. *Culicoides stellifer* (Coquillett) and *C. insignis* Lutz, putative EHDV vectors in the southeastern USA [24], also develop in similar habitats [22]. The objective of this study was to evaluate the potential to use stable isotope labeling of larval *C. sonorensis* in a natural mud substrate under laboratory conditions.

## Methods

Mud was collected from wastewater ponds (known to harbor immature *C. sonorensis*) at a dairy in San Jacinto, CA, USA, in June 2014, and frozen at − 20 °C to kill any preexisting insects. On 1 August 2014, mud was thawed and homogenized by mixing, and 200 ml of mud was added to each of a series of 450 ml clear plastic deli containers, and the same mud was used for both treatment and control replicates. Mud was formed into a gentle “bank” by tapping the bottom edge of the containers against the lab bench. The development substrate (i.e. mud) was allowed to settle briefly (~ 30 min), and then 50 ml of enriched water containing either a “high” or “low” dose of ^15^N-labeled potassium nitrate (KNO_3_; ^15^N, 99 atom%; Cambridge Isotope Laboratories, Inc., Andover, MA, USA) or ^13^C-labeled glucose (U-^13^C_6_, 99 atom%; Cambridge Isotope Laboratories, Inc., Andover, MA, USA) was added to each container such that approximately 1/3 of the mud “bank” was submerged to replicate field conditions. For the high-dose and low-dose treatments, 6 or 2 mg of KNO_3_ or U-^13^C_6_ respectively, were dissolved in 1 l deionized water. Low doses were similar to those used in previous laboratory mosquito labeling studies [21], and because it was unknown whether this dose would be sufficient to label *C. sonorensis* in mud habitats, a high dose of three-times the low dose was selected. Control replicates received 50 ml of deionized water. Ten replicates of each treatment (^15^N-high, ^15^N-low, ^13^C-high, ^13^C-low, control) were used. No additional marked solution was added to the containers after the initial set-up, but additional deionized water was added as-needed throughout the experiment to maintain constant water levels.

Insects used for the study came from an established southern California colony of *C. sonorensis* (Van Ryn strain) maintained at the University of California, Riverside. *Culicoides sonorensis* eggs were laid on moist filter paper on 24 June 2014, and were stored at 4 °C until the start of the experiment (1 August 2014). A small piece of the filter paper with ~ 150–200 eggs was placed 2 cm above waterline in each container immediately after water (control or enriched) was added to the container. The containers were then covered with plastic lids with holes poked in them for air flow. Containers were randomly distributed on a window shelf where they received natural, but not direct, sunlight, and were rotated periodically to account for differences in light exposure. Fluorescent lights were also positioned on both sides of the shelf on a 12:12 h light:dark photoperiod to provide additional light, and mimic typical colony maintenance conditions. The temperature in the laboratory was approximately 23 °C. Containers were checked every 1–3 days for emerged adults. When adults were observed in the containers, they were aspirated into microcentrifuge tubes through access holes cut in the side of the containers, pooled by treatment, and stored at − 20 °C for processing. Each treatment used a dedicated aspirator to prevent cross-contamination. The number of emerged adults per collection day from each container was recorded. Day of emergence was recorded as the number of days since eggs were added to containers. Emergence was considered complete for a given replicate after 3 days with no emergence. Periodically, the mud was gently disturbed by raking the surface with a treatment-specific glass pipette in order to re-suspend nutrients into the water to ensure that microorganisms were present to serve as food for *C. sonorensis* larvae.

Emerged midges from treatment replicate containers were pooled before being processed for the isotope analysis. *Culicoides* samples were analyzed for isotope enrichment at the Texas A&M University Stable Isotope Geosciences Facility using a Thermo Fisher Scientific Delta V Advantage with Flash EA Isolink attached to a ThermoFinnigan Conflo IV isotope ratio mass spectrometer (IRMS). Insects were pooled in groups of 2–25 individuals by treatment (isotope and dose) for analysis, and 15–17 pools per treatment were analyzed (Additional file 1: Table S1). To attempt to determine whether ^13^C or ^15^N labeled *Culicoides* could be detected in mixed pools of unenriched individuals, a small number of pools of six labeled and control insects were also tested. Pools were spiked with either one or three ^15^N-high or ^13^C-high enriched midges, and two replicates of each pool were analyzed (Additional file 1: Table S2). Pools of insects were placed in tin capsules stored in 96-well plates, and insects were dried at 50 °C for 24 h, after which capsules were crimped shut [25], before being analyzed for isotope abundance. Briefly, each sample (tin capsule) is combusted with pure O_2_ at 1020 °C. The combusted sample passes through a reactor bed containing chromium oxide and cobaltous oxide. The resulting oxidized sample gases are then passed through a second, reducing reactor filled with reduced copper wire and held at 650 °C. This step is required to convert the nitrogen oxides generated in the oxidation reactor to N_2_ gas amenable for IRMS analysis. Water generated by combustion is trapped using an in-line bed of anhydrous magnesium perchlorate. Subsequently, the sample gases are chromatographically separated at 50 °C before traveling to the open split of the Conflo III and being introduced into the IRMS.

The peak areas of sample mass-to-charge ratios 28 (N_2_) and 44 (CO_2_) of a combusted sample are converted to total mass of nitrogen and carbon, respectively, using an intra-run calibration. This calibration consists of a methionine standard prepared at 5 masses ranging from 0.1 mg to 3 mg. The resulting peak areas from these standard analyses are regressed against the known amount of nitrogen and carbon present in each of the masses of methionine utilized in the calibration, a relationship that is highly linear. This calibration is then applied to the peak areas of unknown samples within the run, allowing calculation of their total nitrogen and carbon content. Raw sample δ^15^N and δ^13^C measurements are converted to the Air and Vienna Pee Dee Belemnite (VPDB) isotopic scales, respectively, through an intra-run, two-point calibration of ~ 1 mg of l-glutamic acid standards with known isotopic values. The l-glutamic acid standards utilized are USGS 40 (δ^15^N = − 4.52‰ Air, δ^13^C = − 26.39‰ VPDB) and USGS 41 (δ^15^N = 47.57‰ Air, δ^13^C = 37.63‰ VPDB). Internal laboratory standards, at least one of which is similar to the sample matrix, are utilized as internal checks of the accuracy and precision of the calibrations. Powdered rice was used as the standard in this study (δ^15^N = 1.0‰ Air, δ^13^C = − 29.1‰ VPDB) with an internal uncertainty of ± 0.2‰ for both δ^15^N and δ^13^C (1 sigma).

Data were analyzed using R (version 3.4.0). Statistical differences in the mean day of emergence, median day of emergence, and the mean number of emerged adults per treatment were analyzed using analysis of variance (ANOVA) followed by Tukey’s honestly significant difference (HSD) test for means separation. Bonferroni’s correction for multiple comparisons (α = 0.005) was used. The effect of pool size on δ^13^C and δ^15^N was analyzed using generalized linear models (GLM) using ‘Treatment’ and ‘Pool’ as fixed factors and δ^13^C or δ^15^N as the response variable. Differences in the mean δ^13^C or δ^15^N by treatment were analyzed using a Kruskal-Wallis rank sum test followed by Dunn’s test for means separation (*dunn.test* package) [26]. Differences in the amount of isotope incorporated into *C. sonorensis* tissues by isotope treatment were determined by calculating the percent change in δ^13^C and δ^15^N for each treatment replicate compared to the mean δ^13^C or δ^15^N of the unenriched controls. The mean percent change for each treatment was then analyzed by ANOVA, followed by Tukey’s HSD. For mixed pools, a natural isotope abundance baseline for groups of six *C. sonorensis* was calculated from the mean δ^13^C and δ^15^N of the mixed pools spiked with midges enriched with the opposite element (i.e. the δ^13^C for ^15^N labeled mixed pools and *vice versa*). Because the amount of other elements in the sample is not affected by enrichment, this allowed us to generate an estimate of natural ^13^C and ^15^N abundance for pools of six midges. The δ^13^C and δ^15^N of the ^13^C- and ^15^N-spiked mixed pools was then compared to this baseline natural abundance. Labeled *Culicoides* were considered detectable in the mixed pool if the δ^13^C /δ^15^N value for that pool was at least three standard deviations above the mean of the natural abundance baseline [27].

## Results

The first emerged adult midge was recorded on day 23 in a ^13^C-low dose replicate. Midges had begun to emerge in all treatments by day 32, though there was variation among replicates. Midges first emerged in each cup on days 28–46 for controls, days 32–42 for ^13^C-high, days 23–43 for ^13^C-low, days 29–46 for ^15^N-high, and days 25–43 for ^15^N-low. The average day of emergence was significantly later in enriched treatments than in controls (Table 1) (*F*_(4, 3222)_ = 49.1, *P* < 0.0001). Both ^15^N treatments and the ^13^C-high dose treatment had the latest average emergence date. Emergence in the ^13^C-low dose treatment was earlier than the other enriched treatments (*P *< 0.0001), but still later than controls (*P* = 0.0004). However, the average median day of emergence across replicates within a treatment did not differ between treatments. Although the number of emerged adults varied among replicates for all treatments (ranging between 19–129), there was no significant difference among treatments (Table 1).

**Table 1:** Effect of stable isotope enrichment on *C. sonorensis* emergence

Treatment	Average day of emergence (Mean ± SD)	Median day of emergence (Mean ± SD)	No. of emerged adults (Mean ± SD)	No. of emerged adults (Range)
^13^C-High	55.0 ± 9.00^a^	55.5 ± 5.66^a^	71.4 ± 24.1^a^	47–129
^13^C-Low	52.5 ± 9.37^b^	53.4 ± 6.04^a^	70.5 ± 20.2^a^	37–100
^15^N-High	56.4 ± 9.28^a^	56.9 ± 3.50^a^	47.5 ± 22.8^a^	23–77
^15^N-Low	55.9 ± 10.1^a^	57.8 ± 4.61^a^	60.4 ± 25.8^a^	25–99
Control	50.5 ± 7.23^c^	51.3 ± 2.58^a^	66.9 ± 30.3^a^	19–110

*Note*: Superscript letters represent significant differences between treatments in a column (*P* < 0.005)

*Abbreviation*: SD, standard deviation

**Fig. 1:**
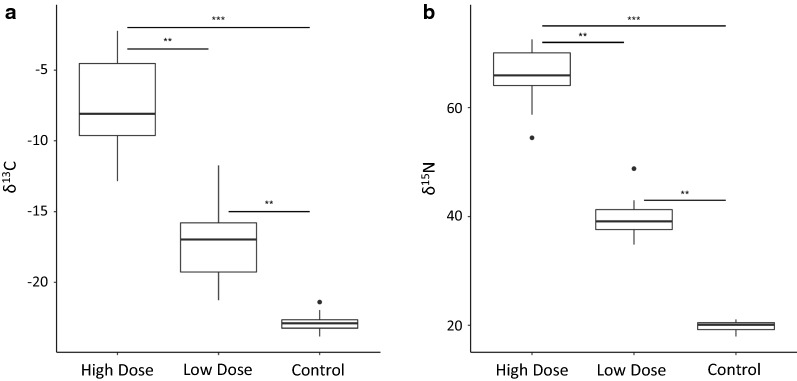
*Culicoides* stable isotope enrichment with high and low doses of ^13^C and ^15^N. Mean δ^13^C (**a**) and δ^15^N (**b**) of pools of *C. sonorensis* adults enriched with high or low isotope doses compared to unenriched controls. Hinges represent upper and lower quartiles, dots represent outliers. **P < 0.01, *** *P *< 0.001

The number of midges in a pool from the same treatment did not affect the δ^13^C (*R*^2^ = 0.92, *P* = 0.77) or the δ^15^N (*R*^2^ = 0.98, *P* = 0.09) values, and pools of two midges had similar delta values as pools of 25 midges. All replicates of various sized pools from the same treatment were therefore combined for further analysis. Mean δ^13^C and δ^15^N values for ^13^C and ^15^N-enriched midge pools were significantly greater than unenriched controls (^13^C: *χ*^2^ = 41.6, *df* = 2, *P* < 0.0001; ^15^N: *χ*^2^ = 41.8, *df* = 2, *P *< 0.0001) (Fig. 1), indicating that these insects had incorporated enough of the isotopes in their tissues during development to make them detectable above background levels. Both the ^13^C and ^15^N-high dose treatments also had significantly higher δ^13^C and δ^15^N values than the low-dose treatments (*P *≤ 0.003). The mean δ^13^C and δ^15^N for the unenriched, control pools was − 22.9‰ and 19.8‰, respectively. The δ^13^C and δ^15^N values for ^13^C and ^15^N low-dose treatment pools were an average (± SD) of 24.3 ± 11.2% and 49.8 ± 3.89% higher than controls, respectively. The δ^13^C and δ^15^N values for ^13^C and ^15^N high-dose treatment pools were an average (± SD) of 68.2 ± 13.7% and 69.7 ± 2.47% higher than controls, respectively. The percent increase in isotope enrichment compared to controls was significantly different across all treatments (*F*_(3, 60)_ = 83.0, *P* < 0.0001), except for the ^13^C and ^15^N high-dose treatments, which did not differ from each other. For mixed pools of six midges, the natural abundance baseline δ^15^N was 20.5 ± 0.35‰, and the natural abundance baseline δ^13^C was − 23.4 ± 0.30‰. The δ^15^N of the ^15^N-mixed pools ranged from 24.8‰ (1 enriched specimen with 5 unenriched) to 40.0‰ (3 of 6 enriched). The δ^13^C of the ^13^C-mixed pools ranged from − 21.6‰ (1 of 6 enriched) to − 15.1‰ (3 of 6 enriched). The ^15^N-mixed pool with the lowest δ^15^N was twelve standard deviations above the natural abundance mean, and the ^13^C-mixed pool with the lowest δ^13^C was six standard deviations above the natural abundance mean.

## Discussion

This study provides support that stable isotope labeling can be an effective means to mark immature *Culicoides* in larval habitat for mark-capture studies of adult dispersal. While previous studies have shown that aquatic habitats can be enriched with stable isotopes to label developing insects, here we show proof of concept that insects developing in semi-aquatic habitats can be labeled in the same way. To the best of our knowledge, this study also represents the first time that *Culicoides* biting midges have been successfully labeled using stable isotope enrichment. Compared to other insects that have been targeted in previous stable isotope labeling studies, *Culicoides* are small, and it was unknown whether a single midge would contain enough isotope to fall within the instrument’s detection limits, or whether a single enriched midge could be detected in a pool of unenriched individuals.

In order to determine how many midges would need to be pooled in order to get a quantitative isotopic measurement, we analyzed pools of ^13^C and ^15^N-enriched *C. sonorensis* ranging between 2–25 individuals. There was no significant effect of pool size on δ^13^C or δ^15^N, indicating that accurate readings can be achieved with very small pool sizes (potentially as small as a single midge) when using the methods and instrumentation described here. Additionally, larger pool sizes did not interfere with isotopic measurement accuracy, as had previously been shown for pools of ten *Culex pipiens* (Forskål) [21]. A small number of midges were dried and weighed in pools of 4–9 using a Sartorius CP2P microbalance (Sartorius Corporation, Edgewood, NY, USA), and the mean weight of a single midge was determined to be ~ 40 µg. This is a substantially lower sample mass threshold for the accurate determination of δ^13^C or δ^15^N values than used in previous stable isotope labeling studies, and supports the potential to use isotopic labeling as part of a mark-capture study of *Culicoides* adult dispersal.

Both high and low dose treatments for ^15^N- and ^13^C- labeled *C. sonorensis* were sufficient to uniquely enrich midges above the natural isotope abundance levels of control specimens, though even pools of 20–25 midges from high-dose treatments did not reach the same δ^13^C or δ^15^N levels compared to single mosquitoes enriched with the same doses [21, 28]. The highest δ^15^N we recorded for a ^15^N-enriched pool of *C. sonorensis* was 72.6‰ and the highest δ^13^C we recorded for a ^13^C-enriched pool was − 2.23‰. For comparison, a previous study enriching *Cx. pipiens* resulted in δ^15^N and δ^13^C values of 514–824‰ and 73–603‰, respectively [21]. It is possible that assimilation of these enriched elements is less efficient in *Culicoides*. Alternatively, given that bioaccumulation of the stable isotopes through a microbial community which is fed upon by larvae is the most likely mechanism of enrichment, we might not have achieved efficient bioaccumulation in these simulated mud substrate habitats. We used mud from the field which had been frozen to kill any wild insects present. The microbial community of this frozen and thawed mud was unknown, and using fresh mud, with an unaltered microbiome, might have improved delivery to the insects. Alternatively, adding the enriched water to the mud containers several days before adding eggs might also have increased enrichment in the adult *Culicoides* by allowing more time for the isotopes to be fully incorporated in the substrate prior to larvae hatching.

Because of the low capture rate typical of mark-capture studies, the ability to detect a single enriched individual in a pool of unenriched midges is critical for the successful use of stable isotope labeling for *Culicoides* studies. For proof of concept, we tested a small number of mixed pools of labeled and control midges to determine whether these pools would be detectable as “enriched.” These mixed pools had δ^13^C and δ^15^N values well above the natural isotope abundance baseline of unenriched pools, even when only one enriched individual was present, though variation would likely be greater in field collected samples, and single midges may be less detectable in larger pool sizes than tested in this study. Future enrichment studies of *Culicoides* should consider a higher dose of stable isotopes or deliver a dose repeatedly over time to achieve higher δ^15^N and δ^13^C, which would improve the ability to detect a single marked individual in a pool of natural abundance specimens.

Enrichment of the larval habitat did not appear to negatively affect *C. sonorensis* survival, but did delay the average adult emergence time by approximately five days. Emergence was the least affected in the ^13^C-low dose treatment. The delayed emergence of enriched midges may not have a significant impact on field collections in *Culicoides* mark-capture studies for dispersal as long as traps are set for a sufficient amount of time, but should be considered if the study objectives include questions about development. Previous studies comparing ^15^N and ^13^C enrichment in mosquitoes showed a trend towards higher levels of nitrogen integration in tissues compared to carbon, potentially due to the use of nitrogen-rich food sources [28]. We found that in low-dose treatments, more ^15^N was integrated into *C. sonorensis* tissues than ^13^C; a ~ 50% increase compared to a ~ 24% increase. However, when isotope concentrations were increased, there was no difference in the percent increase in δ^15^N or δ^13^C, suggesting that at higher doses there is no benefit to using one isotope over the other.

Stable isotope labeling has the benefit of allowing researchers to positively connect captured, adult midges to specific and known larval development sites. Immunomarking is the only other mark-capture technique that allows for this type of data collection. Both techniques potentially allow for differentiation of multiple marked sites by using either different proteins or different atomic elements. Immunomarking may be more accessible to most entomology laboratories, as samples are analyzed using a protein-specific ELISA, while stable isotope labeling requires access to specialized facilities. Costs for processing stable isotope enriched samples vary by institution, though the cost is higher than processing immunomarked samples by ELISA. Using the commercially available ovalbumin ELISA from Sanders & Carpenter [29] costs ~ 6.20 USD/sample, and the cost/sample for isotopic analysis in this study was 9.00 USD. However, stable isotope labeling has several advantages over immunomarking which may make it a more desirable method in some instances. Because immunomarking involves an insect coming in contact with the protein marker and picking it up on its body, there is the potential for unmarked individuals to become contaminated with the marker in a trap, and for insects which did not develop in the marked habitat to become marked simply by contacting the surface [14]. Additionally, *Culicoides* immunomarking has thus far only been studied in manure-developing species [14, 29], and it is unknown whether the technique could be applied to semi-aquatic developing species, like *C. sonorensis*. Stable isotope labeling cannot be transferred between individuals, individuals can only be marked by developing in enriched habitats, and it is compatible with aquatic and semi-aquatic habitats.

In this feasibility study, we labeled *Culicoides* developing in small, contained substrates in the laboratory environment. Previous field trials labeling mosquitoes with stable isotopes focused on enrichment of smaller container habitats (e.g. catch basins, plastic tubs) [21, 25, 28]. One limitation of the present study is that natural *Culicoides* habitats are often larger than previously studied mosquito habitats, and not artificially contained, like dairy wastewater ponds. These types of habitats would likely require dramatically more isotopic material in order to enrich the specimens than used in container-breeding mosquito studies. A potential solution to this would be to target smaller, highly productive *Culicoides* sites, or construct a more concentrated experimental field habitat for enrichment. Although we did not measure isotope retention in older individuals in this study, future work should determine whether enrichment is lifelong in *Culicoides* spp.

## Conclusions

*Culicoides sonorensis* can be successfully labeled with ^15^N and ^13^C stable isotopes when the larval habitat is enriched with either a low or a high dose of the isotope. Elevated levels of stable isotope were detected in pools of 2–25 individuals for both low and high doses. A single enriched midge can potentially be detected in a small pool of unenriched midges, though further work is needed to determine the limit of detection using this method. Stable isotope labeling shows promise for future *Culicoides* mark-capture adult dispersal studies in the field.

## Supplementary information

**Additional file 1: Table S1.** Pools and pool sizes of emerged adult *C. sonorensis* analyzed for stable isotope enrichment. **Table S2.** Mixed pools of labeled and control *C. sonorensis* analyzed for stable isotope enrichment to determine whether labeled midges were detectable in groups of unlabeled midges.

## Data Availability

Data are available upon request.
