# Supplementary material for: Laboratory evaluation of stable isotope labeling of Culicoides (Diptera: Ceratopogonidae) for adult dispersal studies
Source: Parasit Vectors. 2019 Aug 22;12:411. doi: 10.1186/s13071-019-3671-9 (PMC6704667; doi:10.1186/s13071-019-3671-9)
Supplement: Supplementary file 1 — Additional file 1: Table S1. Pools and pool sizes of emerged adult C. sonorensis analyzed for stable isotope enrichment. Table S2. Mixed pools of labeled and control C. sonorensis analyzed for stable isotope enrichment to determine whether labeled midges were detectable in groups of unlabeled midges. [file 13071_2019_3671_MOESM1_ESM.docx]

**Additional file 1: Table S1.** Pools and pool sizes of emerged adult *C. sonorensis* analyzed for stable isotope enrichment

| Treatment | Pool Sizes Tested | Pools Tested/Size | Total Pools | Total Insects Tested |
| --- | --- | --- | --- | --- |
| Control | 2 | 2 | 16 | 145 |
|  | 4 | 2 |  |  |
|  | 5 | 1 |  |  |
|  | 6 | 2 |  |  |
|  | 8 | 2 |  |  |
|  | 10 | 4 |  |  |
|  | 15 | 1 |  |  |
|  | 20 | 1 |  |  |
|  | 25 | 1 |  |  |
| ^13^C-Low | 2 | 2 | 15 | 135 |
|  | 4 | 2 |  |  |
|  | 5 | 1 |  |  |
|  | 6 | 2 |  |  |
|  | 8 | 2 |  |  |
|  | 10 | 3 |  |  |
|  | 15 | 1 |  |  |
|  | 20 | 1 |  |  |
|  | 25 | 1 |  |  |
| ^13^C-High | 2 | 2 | 17 | 165 |
|  | 4 | 2 |  |  |
|  | 5 | 1 |  |  |
|  | 6 | 2 |  |  |
|  | 8 | 2 |  |  |
|  | 10 | 4 |  |  |
|  | 15 | 1 |  |  |
|  | 20 | 2 |  |  |
|  | 25 | 1 |  |  |
| ^15^N-Low | 2 | 2 | 15 | 135 |
|  | 4 | 2 |  |  |
|  | 5 | 1 |  |  |
|  | 6 | 2 |  |  |
|  | 8 | 2 |  |  |
|  | 10 | 3 |  |  |
|  | 15 | 1 |  |  |
|  | 20 | 1 |  |  |
|  | 25 | 1 |  |  |
| ^15^N-High | 2 | 2 | 17 | 165 |
|  | 4 | 2 |  |  |
|  | 5 | 1 |  |  |
|  | 6 | 2 |  |  |
|  | 8 | 2 |  |  |
|  | 10 | 4 |  |  |
|  | 15 | 1 |  |  |
|  | 20 | 2 |  |  |
|  | 25 | 1 |  |  |

**Additional file 1: Table S2.** Mixed pools of labeled and control *C. sonorensis* analyzed for stable isotope enrichment to determine whether labeled midges were detectable in groups of unlabeled midges

| Treatment | No. Labeled/  No. Control | Replicates | δ Values |
| --- | --- | --- | --- |
| ^13^C-High | 1/5 | 2 | -21.6, -20.5 |
|  | 3/3 | 2 | -18.7, -15.1 |
| ^15^N-High | 1/5 | 2 | 24.8, 26.1 |
|  | 3/3 | 2 | 36.30, 40.0 |
